# Pomegranate extract supplementation improves lung function parameters and IL-35 expression in participants with mild and moderate persistent allergic asthma: A randomized, double-blind, placebo-controlled trial

**DOI:** 10.3389/fnut.2022.1026343

**Published:** 2022-10-18

**Authors:** Zainab Shateri, Seyed Ahmad Hosseini, Farhad Abolnezhadian, Elham Maraghi, Maryam Haddadzadeh Shoushtari, Marzie Zilaee

**Affiliations:** ^1^Student Research Committee, Ahvaz Jundishapur University of Medical Sciences, Ahvaz, Iran; ^2^Nutrition and Metabolic Diseases Research Center, Ahvaz Jundishapur University of Medical Sciences, Ahvaz, Iran; ^3^Nutrition Department, Faculty of Paramedicine, Ahvaz Jundishapur University of Medical Sciences, Ahvaz, Iran; ^4^Division of Immunology and Allergy, Department of Pediatrics, Abuzar Children's Hospital, Ahvaz Jundishapur University of Medical Sciences, Ahvaz, Iran; ^5^Department of Biostatistics and Epidemiology, School of Public Health, Ahvaz Jundishapur University of Medical Sciences, Ahvaz, Iran; ^6^Air Pollution and Respiratory Disease Research Center, Ahvaz Jundishapur University of Medical Sciences, Ahvaz, Iran

**Keywords:** asthma, high-sensitivity C-reactive protein, interleukin-35, lung function, pomegranate

## Abstract

Existing asthma treatments are associated with side effects and limitations, which has led to an interest in alternative and complementary therapies. Given the anti-inflammatory properties of pomegranate, the present study aimed to determine the impact of pomegranate extract supplementation on lung function parameters evaluated through spirometry, high-sensitivity C-reactive protein (hs-CRP), pro-oxidant antioxidant balance, and interleukin 35) (IL-35) in participants with mild and moderate allergic asthma (based on forced expiratory volume in 1 second (FEV_1_) and clinical symptoms). Participants with mild and moderate allergic asthma (*n* = 64) were randomly assigned to two groups: the intervention group, which received two pomegranate extract capsules (500 mg/day), or the control group for eight weeks. Also, the physician prescribed similar drugs to the participants in the study. Independent samples *T*-test and Mann–Whitney U were used to compare the quantitative outcomes between the intervention group and the comparison group. The Wilcoxon test and the paired *T*-test were applied for within-group comparisons. A *p*-value <0.05 was considered significant. At the end of the study, the change levels of IL-35 in the intervention group increased significantly compared to the control group. In terms of the lung function parameters, FEV_1_/ forced vital capacity (FVC) (FEV_1_/FVC) ratio enhanced significantly in the intervention group compared to the control group. Also, the pomegranate extract significantly improved forced expiratory flow 25–75% (FEF_25−75%_), FEV_1_/FVC ratio, and FEV_1_ in the intervention group. No significant changes in FEV_1_ values were observed between the two groups at the end of the study. Also, no significant changes were seen in other indicators. It seems that pomegranate extract can improve lung function parameters and IL-35 expression in mild and moderate allergic asthma.

**Clinical trial registration:**

https://www.irct.ir/trial/45612; identifier: IRCT20200205046384N1.

## Introduction

Asthma is a multifaceted and complicated illness caused by the interaction between environmental exposure, host factors, and genetic susceptibility (1). It is one of the most common chronic respiratory diseases (2), affecting more than 300 million people worldwide (3).

Inflammation, increased airway responsiveness, remodeling alternations, excessive mucus secretions, and reversible airway obstruction are the prominent features of asthma (4, 5). Immune system cells and airway structural cells emit a variety of cytokines and inflammatory mediators, which contribute to airway inflammation in people with asthma (5). Continuous respiratory tract inflammation is accompanied by a mucosal influx of mast cells, T lymphocytes, eosinophils, and the diffusion of lipid mediators and pro-inflammatory cytokines (6). The airways produce a diversity of inflammatory mediators due to exposure to environmental oxidants, including reactive oxygen species (7). Pro-oxidant antioxidant balance (PAB) assessment simultaneously evaluates pro-oxidant load and antioxidant capacity, showing us an indicator of redox status (8).

One of the most prominent indicators of the inflammatory process is C-reactive protein (CRP) (9). The serum high-sensitivity CRP (hs-CRP) levels can represent levels of CRP that are not detectable using usual methods and can assess even mild inflammation in many diseases, including cardiovascular disease and diabetes (10). The association between asthma control and serum levels of hs-CRP in adult patients has been investigated, and the results have been encouraging (11, 12). In addition, interleukin 35 (IL-35) appears to suppress asthma's allergic inflammation (13). Recent evidence suggests the role of IL-35 in the pathophysiology of asthma and considers IL-35 to be a critical factor in regulating cellular relationships, differentiation, and inflammation (13).

Spirometry is one of the lung function tests to evaluate the response of patients with asthma to treatment, and clinical symptoms (14). Among the different variables measured by spirometry test, forced expiratory volume in 1 second (FEV_1_), indicating the severity of airway obstruction, is the most commonly used (14, 15) and also the most reproducible pulmonary function parameter (15). The severity of asthma symptoms is categorized as mild, moderate, and severe, based on FEV_1_ values (14).

The health benefits of pomegranate, as a rich source of polyphenols, in animals and humans have been extensively studied (16–20). It has been found that polyphenols have anti-inflammatory and antioxidant features in the human body (21). According to a study on the impact of ellagic acid (one of the main ingredients of pomegranate) on asthmatic mice, the results showed that ellagic acid had significant anti-inflammatory activity in asthma and might be regarded as a therapeutic agent for allergic asthma (22). In a study that examined the effect of pomegranate juice supplementation on inflammatory markers in patients with diabetes, it was demonstrated that taking 250 mL of pomegranate juice daily for 12 weeks led to a significant reduction in IL-6 and serum hs-CRP levels (23).

The significant increase in asthma incidence in the last few decades and the geographical variation in both the magnitude of the increases and base prevalence rates confirm the hypothesis that environmental alterations are involved in the present asthma epidemic (24).

The incidence of asthma in Iran is reported to be higher than the global and regional average due to the transition to urban and industrial life, specific climatic conditions, and industrial pollution in the country. According to the Iranian Asthma and Allergy Association, the prevalence of asthma in Iran is between 5 and 15% (25), while in Ahvaz, the number of people with asthma is more than twice the national average (26).

In general, given that asthma exerts a significant effect not only on the quality of life related to a person's health status but also through absenteeism in the workplace and early retirement, it will impose high costs on the economy and society (27). Also, existing asthma treatments are associated with side effects and limitations, which has led to an interest in alternative and complementary therapies (28). Therefore, given the anti-inflammatory properties of pomegranate, the present study aimed to determine the impact of pomegranate extract on the lung function parameters evaluated through spirometry, hs-CRP, PAB, and IL-35 in participants with mild and moderate allergic asthma.

## Methods

### Design of trial

The current study was approved by the Ahvaz Jundishapur University of Medical Sciences ethics committee (Registration No. IR.AJUMS.REC.1398.905) and registered at the Iranian Registry of Clinical Trials (IRCT20200205046384N1). The present study applied a randomized controlled trial using a double-blind design.

The formula for comparing averages with β = 0.2, α = 0.05, S_1_ = 29.8, S_2_ = 0.71, μ_1_ = 16.13, μ_2_ = 0.9 was applied to compute the sample size considering the PAB variable levels based on a previous study (29):


(1) $$\begin{array}{l} N=\left({\left[Z_{1-\text{α}/2}+Z_{1-\text{β}}\right]}^{2}\left[S_{1}^{2}+S_{2}^{2}\right]\right)/{\left({\text{μ}}_{1}-\;{\text{μ}}_{2}\right)}^{2} \end{array}$$

According to the formula mentioned above, at least 30 patients were required to participate in the intervention and control groups. Assuming 15% removal or violation of protocols, 35 people were assigned to each group. The participants signed an informed consent form at the beginning of the study. The researchers randomly classified participants using computer-generated blocked randomization based on asthma severity and clinical symptoms (prepared by a biostatistics specialist). To follow the double-blind scheme, a person was asked to number the capsule bottles. Each participant was given a code based on the permuted block. Each participant completed a questionnaire on demographic information and personal characteristics, such as age and age of onset of asthma, at the beginning and end of the study. Also, participants' weight, type, and dose of the drugs used were recorded at the beginning and end of the study. Height was also measured at the beginning of the study. Furthermore, participants in both groups were asked not to alter their physical activity and energy received throughout the study. Physical activity was assessed at the beginning and end of the study through the international physical activity questionnaire. Allergic asthma was confirmed by measuring the total serum immunoglobulin E (IgE) levels. Since the serum levels of IgE are more reliable than other tests for allergic asthma, such as a skin prick test, we used the serum levels of IgE to confirm allergic asthma (30). The serum levels of IgE ≥ 30 international units (IU) are known as allergic patients (31). Therefore, individuals with serum IgE levels ≥ 30 participated in the present study. The questionnaires were completed, and blood samples were taken at the beginning and end of the study (after 8 weeks). Participants were asked not to consume pomegranate juice, pomegranate paste, and pomegranate-containing products throughout the study. They were also asked to avoid certain foods if they were allergic to them, not to eat processed foods such as sausages, and not to take nutritional supplements. Moreover, to evaluate the participants' diet (total calories and received macronutrients) at the beginning and end of the study, the 24-h dietary recall on 3 days (2 weekdays and 1 weekend day) was recorded. Participants should not suffer from any disease other than asthma and should not take any medication other than asthma medications. The physician prescribed similar drugs to the participants in the study.

Regular use of the capsules was followed up by a phone call, and the participants were asked to return the capsule bottles at the end of the study.

### Participants

The research participants comprised 30 men and 40 women, ranging from 18 to 65 years. Participants with persistent allergic asthma were categorized into two groups (mild and moderate) based on their severity (FEV_1_ ≥ 60 %) and clinical symptoms using the Global Initiative for Asthma (GINA) diagnostic criteria for asthma by asthma and allergy specialist. In mild persistent asthma, symptoms are experienced more than once a week and less than once a day. The disease's symptoms may affect the patient's activity and sleep. Experiencing nocturnal symptoms occurs more than twice a month, and FEV_1_ is ≥ 80%. In moderate persistent asthma, symptoms are experienced daily and may affect the patient's activity and sleep. Experiencing nocturnal asthma symptoms occurs more than once a week, and FEV_1_ is 60–80% (14).

Participants were selected from asthma and allergy center in Ahvaz, Iran. A description of the trial was given to the participants, and their written consent was obtained to participate in the study. The inclusion criteria of the research were people aged 18–65 years with persistent allergic asthma (mild and moderate), IgE ≥ 30 IU, and body mass index below 30 kg/m^2^. We did not include unwillingness, lactation, pregnancy, taking mineral/multivitamin supplements during the last 2 months, malignancy, smoking, diabetes, other pulmonary diseases, and autoimmune diseases.

### Intervention

The pomegranate extract capsule and the placebo (rusk powder) were prepared by the Institute of Medicinal Plants, Karaj, Alborz Province, Iran. The pomegranate extract capsule and the placebo were identical in size and color. Each capsule of pomegranate extract (consumed twice a day for 8 weeks) contained 250 mg of pomegranate seed extract, 2.1 μg of ellagic acid, 118.4 μg of punicalagin alpha, and 53 μg of punicalagin beta (measured using the high-performance liquid chromatography method). Ghavipour et al. showed that consuming 250 mg of pomegranate extract twice a day for 8 weeks reduced oxidative stress and some blood biomarkers of inflammation in patients with rheumatoid arthritis (20). Therefore, in the study, we applied the same duration of follow-up in both study arms.

### Outcomes

The measurement of PAB, hs-CRP, IL-35, and spirometry tests involved the primary outcomes of this research at the beginning and the end of the 8th week.

### Blood sample collection

At the beginning of the study and the end of the 8th week, venous blood samples (6 mL) were taken from all participants. The centrifuged blood samples were stored at – 80°C in a freezer.

### Laboratory analyses

IL-35 concentration and serum hs-CRP levels were measured by the ZellBio enzyme-linked immunosorbent assay (ELISA) kit (made in Germany) and the CRP high-sensitive ELISA (product code: DM E-4600, brand: LDN), respectively.

### PAB assessment

A corrected PAB was used based on a method explained in a previous study (32). To prepare a standard solution, different ratios of 250 μM of hydrogen peroxide (0–100%) were mixed with uric acid (3 mM) in NaOH (10 mM). Tetramethylbenzidine (TMB) powder (60 mg) was dissolved in dimethyl sulfoxide (DMSO) (10 mL). To ready TMB cation, TMB/DMSO (400 μL) was mixed in acetate buffer (20 mL, pH 4.5, 0.05 M), and then fresh chloramine T solution (70 μL, 100 mM) was added to this 20 mL. The solution was blended finely and incubated at room temperature (in a dark place) for 2 h. Subsequently, a solution of peroxidase enzyme (25 units) was added to 20 mL of a cationic solution of TMB and distributed as 1 mL before storage at – 20°C. To provide the TMB solution, TMB/DMSO (200 μL) was mixed in acetate buffer (10 mL, 0.05 M, pH 5.8), and by mixing TMB cation (1 mL) with TMB solution (10 mL), the working solution was provided. The working solution was incubated in a dark environment at room temperature for 2 min and then applied instantly. Next, 10 μL of each sample, blank (distilled water) or standard, was blended with the working solution (200 μL) in each well and incubated in a dark environment at 37°C for 12 min. At the end of the incubation, 2N hydrogen chloride (100 μL) was added to each well and assessed by an ELISA reader (450 nm, reference wavelength of 570 nm or 620 nm). A standard curve was obtained from the standard sample values.

Hamidi-Koliakos (HK) units were applied to express PAB values, indicating the percentage of hydrogen peroxide in the standard solution. According to the values acquired from the above-mentioned standard curve, unknown sample values were evaluated.

### Lung function

Asthma is clinically recognized via physical examination, patient's history, and spirometry tests. Spirometry tests are useful tools to assess lung function to diagnose asthma and follow the treatment process of the disease (33). Also, spirometry tests show an accurate picture of the asthma severity and the degree of airway obstruction (15). To evaluate the impact of pomegranate extract on the spirometry tests, forced expiratory flow 25–75% (FEF_25 − 75%_), forced vital capacity (FVC), FEV_1_ and FEV_1_/FVC ratio were evaluated at the beginning and end of the study. Before performing spirometry, the patient's identification was checked, their height without shoes or boots and weight were measured, and their age and sex were recorded. Measurement posture was as follows: the participants should sit straight and put their feet on the ground. Also, they were asked to wear loose clothing. We asked the patients not to use long-acting bronchodilators and other bronchodilators 24 and 8 h before the spirometry test, respectively. A Spirolab spirometry device (MIR Company, Italy) was used to evaluate the lung function parameters.

### Statistical analysis

Statistical analysis was performed using the SPSS software version 22 (SPSS Inc. Chicago, IL, USA). The normality of continuous variables was examined using Shapiro-Wilk's *W*-test. Continuous variables are reported as mean (confidence interval (CI) 95%). Categorical data are expressed as numbers (percentages). Independent samples *T*-test or Mann–Whitney U was used to compare PAB, FEV_1_/FVC ratio, FVC, FEF_25 − 75%_, FEV_1_, IL-35, and serum hs-CRP levels between the intervention group (pomegranate extract supplementation) and comparison group (control), whenever needed. The Wilcoxon test and the paired *T*-test were applied for within-group comparison. Also, the False Rate Discovery approach was performed for correction by multiple comparisons. Moreover, subgroup analysis was done based on asthma severity. A *p*-value <0.05 was considered significant.

## Results

**Figure 1:**
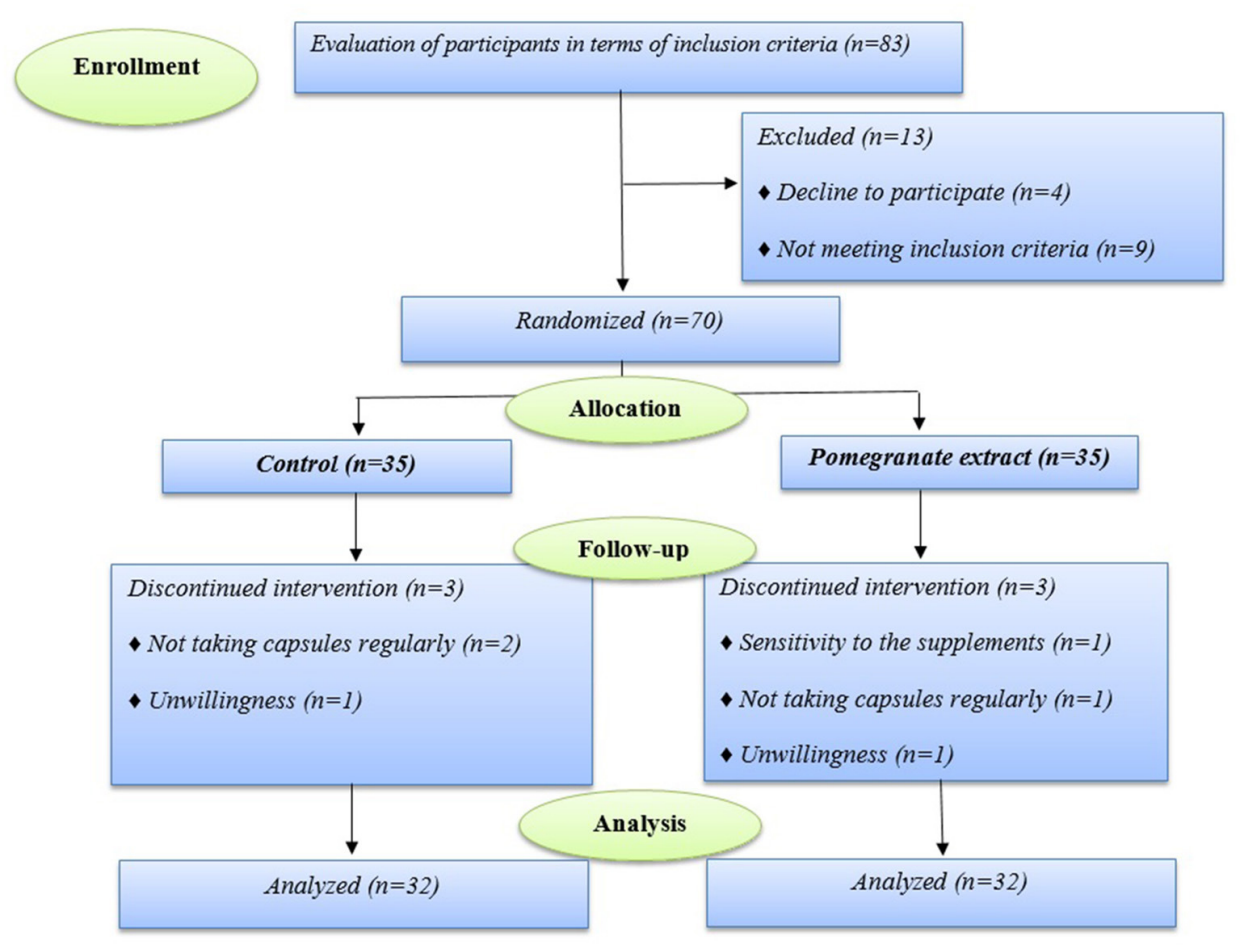
The study flowchart.

During the follow-up period, 64 of 70 participants who enrolled in the trial completed the study. Six of the enrolled ones discontinued the study (in the pomegranate group, one subject because of sensitivity to the supplements, one person did not take capsules regularly, and one person was excluded from the study due to unwillingness, and in the control group, two subject due to did not take capsules regularly, and one person was excluded because of unwillingness) (Figure 1).

### Baseline characteristics

The mean and standard deviation of participants' age was 38.44 ± 11.90 years. In addition, 50% of the participants had mild asthma, and the rest had moderate asthma. Men and women constituted 42.18 and 57.81% of the sample, respectively. No statistically significant differences in dietary and anthropometric variables (data not reported), asthma severity, physical activity, and clinical features were observed between the two groups, as indicated in Table 1 (*p* > 0.05). Furthermore, no significant differences were observed between the two groups regarding the medication taken (Symbicort, Budesonide, Tiova, and Salbutamol spray) and the age of onset of asthma (*p* > 0.05).

**Table 1:** Comparing the initial features between the intervention and control groups.

**Variable**	**Pomegranate (*n* = 32)**	**Control (*n* = 32)**	*P*-value*
**Gender**			
Male N (%)	15 (46.9)	12 (37.5)	0.307
Female N (%)	17 (53.1)	20 (62.5)	
Age (years)	38.94 (34.22, 43.65)	37.94 (34.03, 41.83)	0.740
Age of the onset of asthma (year)	28.72 (22.87, 34.57)	26.88 (20.82, 32.93)	0.651
Asthma severity			0.599
Mild N (%)	16 (50)	16 (50)	
Moderate N (%)	16 (50)	16 (50)	
Systolic blood pressure (mm Hg)			0.916
Diastolic blood pressure (mm Hg)			0.444
Physical activity (MET-min/week)	2257.19 (2143.60, 2370.80)	2127.69 (2042.95, 2212.42)	0.200
Eosinophil (%)	3.24 (2.37, 4.11)	1.75 (1.15, 2.34)	0.002
Neutrophil (%)	59.59 (57.05, 62.13)	58.48 (55.60, 61.37)	0.914
Basophil (%)	0.40 (0.35, 0.45)	0.39 (0.30, 0.47)	0.649
**Symptoms (frequency per day)**			
Daily breath shortness	2.06 (1.17, 2.96)	1.56 (0.64, 2.49)	0.198
Nocturnal breath shortness	0.84 (0.34, 1.35)	1.31 (0.01, 2.61)	0.921
Nocturnal waking up	0.81 (0.25, 1.37)	0.47 (0.14, 0.80)	0.400
Limitation of asthma-related activity	1.18 (0.35, 2.03)	1.60 (0.72, 2.47)	0.222
Salbutamol spray consumption	0.47 (0.10, 0.83)	0.59 (0, 1.27)	0.601
**Infant Feeding N (%)**			
Formula	2 (6.2)	2 (6.2)	0.922
Breast feeding	26 (81.3)	27 (84.4)	
Formula + Breast feeding	4 (12.5)	3 (9.4)	

MET, Metabolic Equivalent Task. For quantitative variables, values are shown as mean (CI 95%). Categorical variables are shown as number (percent).

*Reported *p*-value to compare the research variables between the groups (Mann–Whitney U test and independent sample *T*-test). For qualitative variables, chi-square test and Fisher's exact test were utilized.

### IL-35, hs-CRP, and PAB

**Figure 2:**
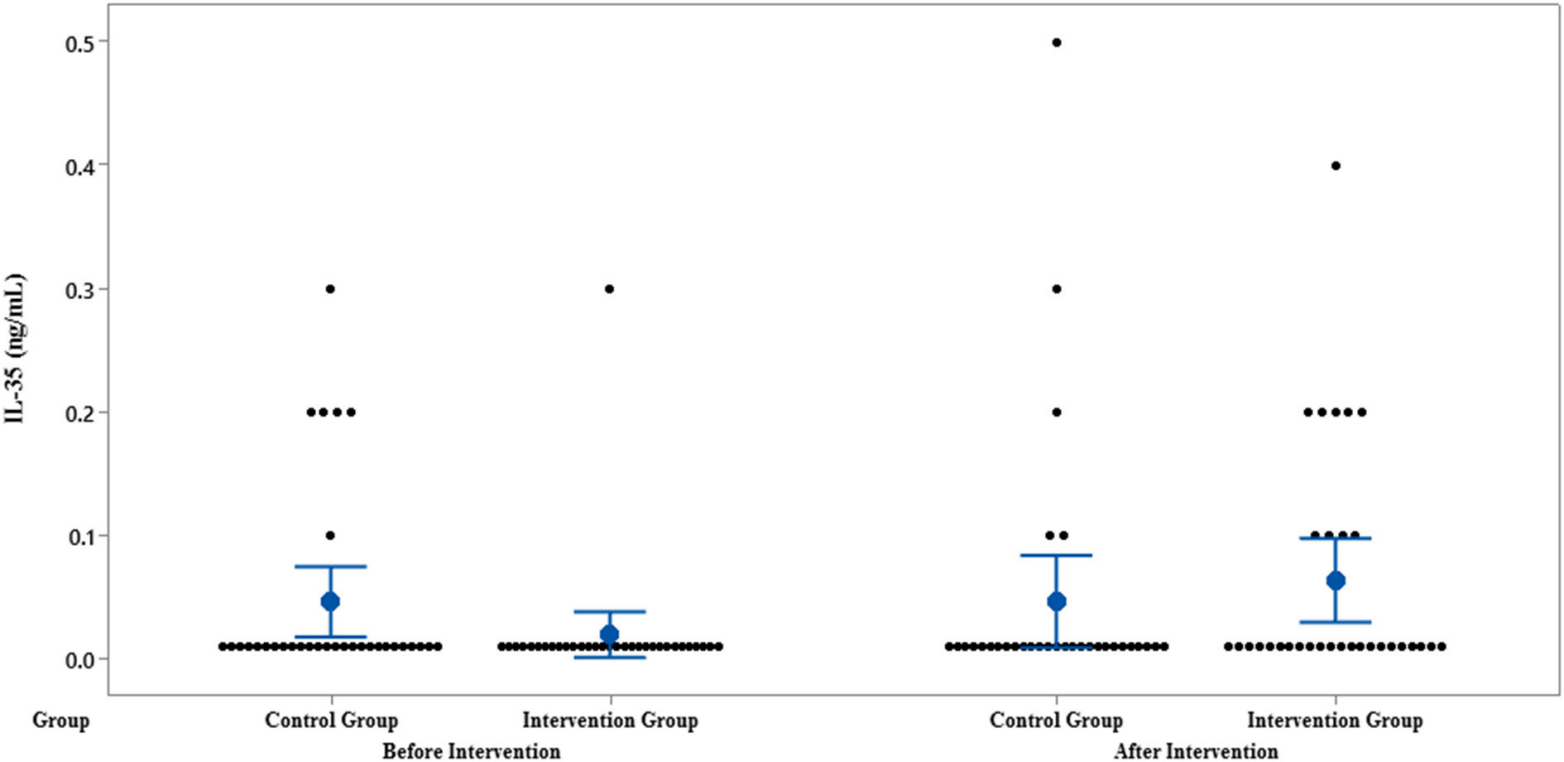
Interval plot of IL-35 pre- and post-intervention with 95% CI for the mean plus the dots for IL-35 values, in each group.

**Figure 3:**
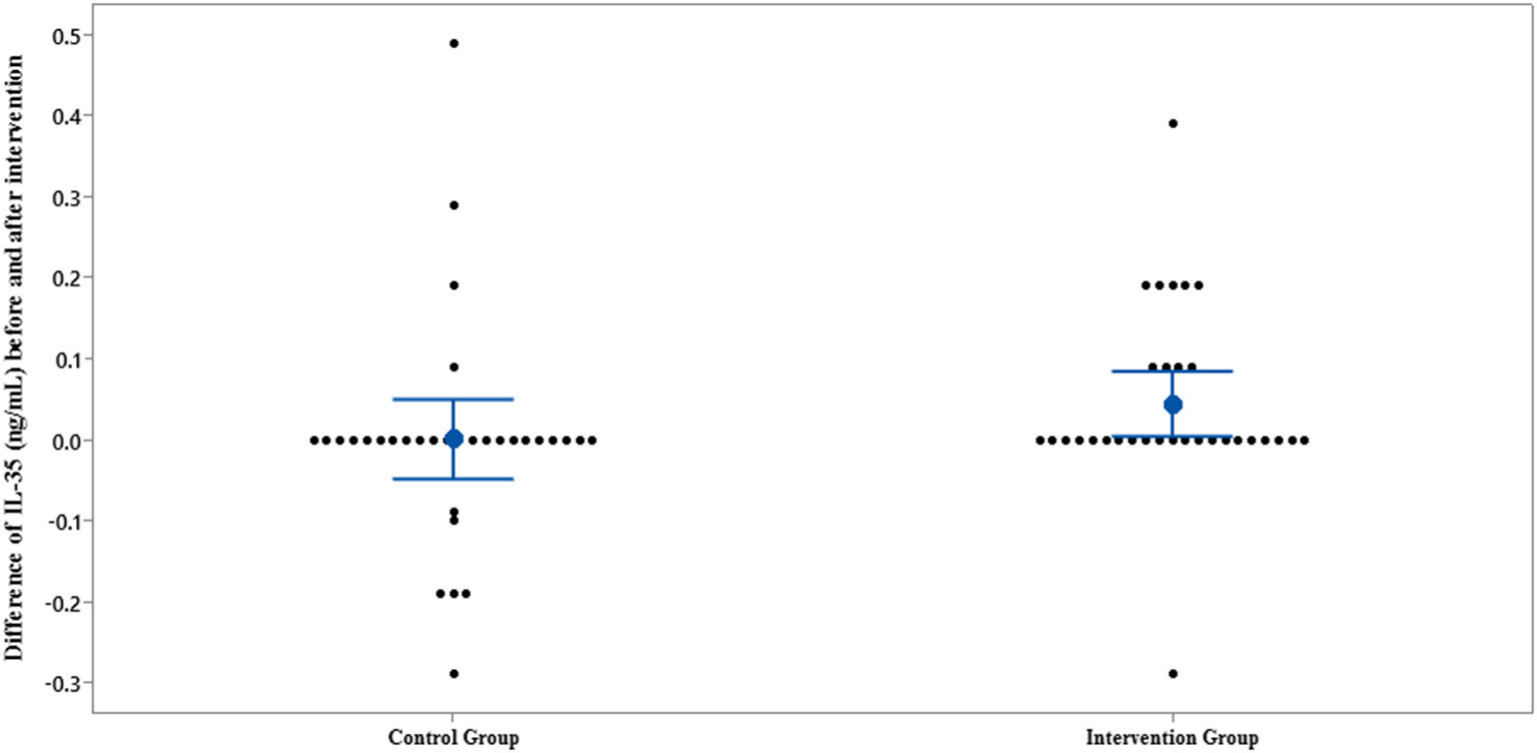
Interval plot of difference between IL-35 pre- and post-intervention with 95% CI for the mean plus the dots for IL-35 values, in each group.

**Figure 4:**
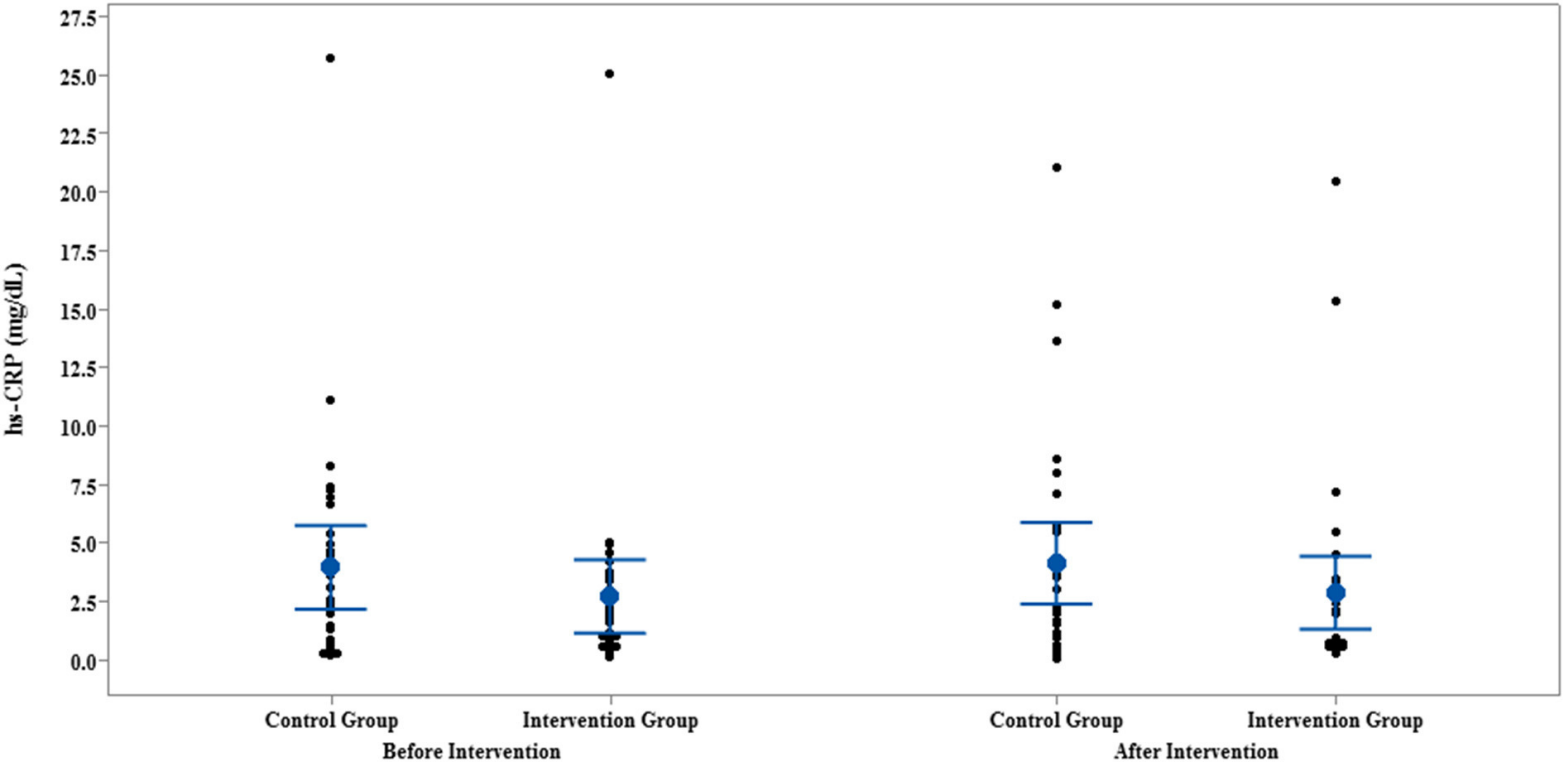
Interval plot of hs-CRP pre- and post-intervention with 95% CI for the mean plus the dots for hs-CRP values, in each group.

**Figure 5:**
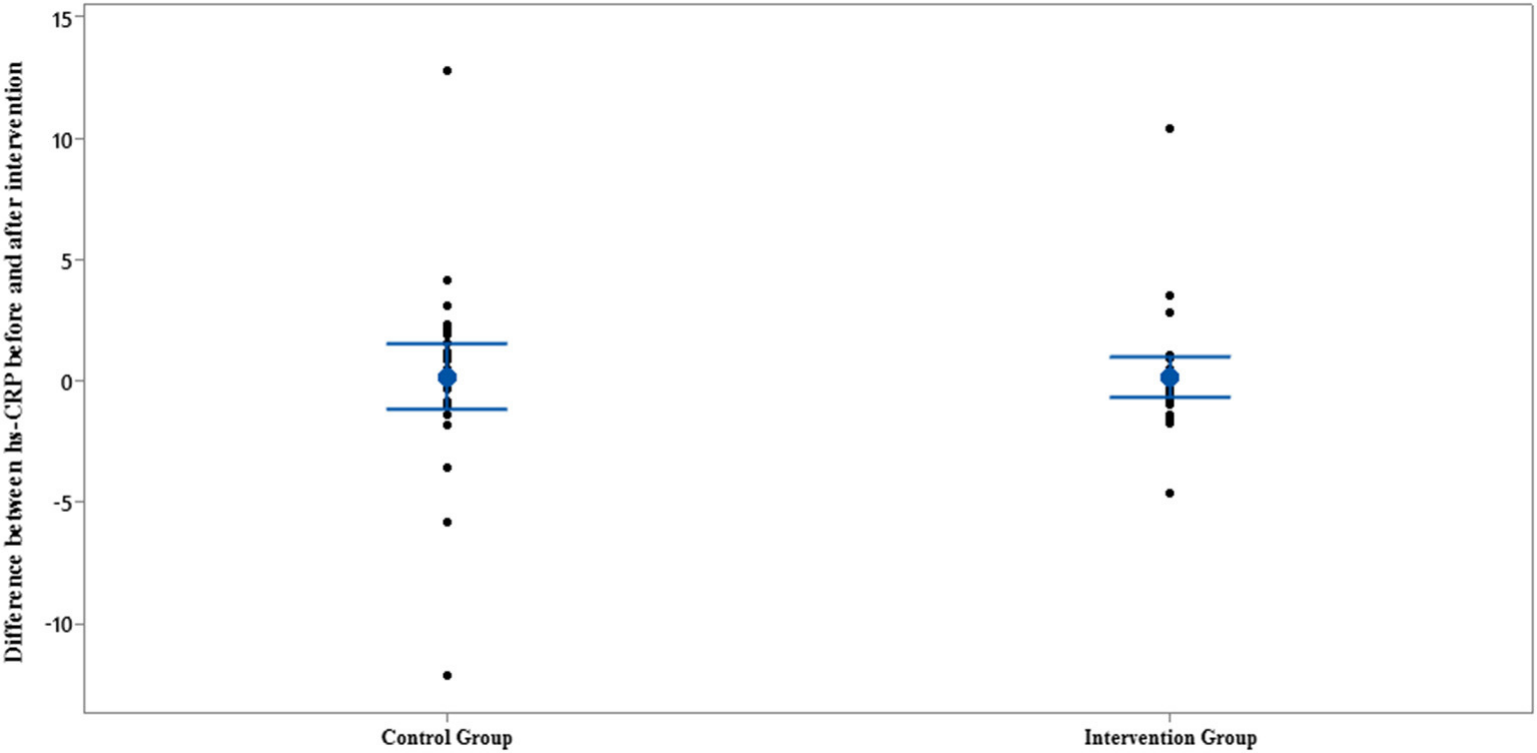
Interval plot of difference between hs-CRP pre- and post-intervention with 95% CI for the mean plus the dots for hs-CRP values, in each group.

The results of IL-35 and serum hs-CRP levels are presented in Figures 2–5 and Table 2. At the beginning of the study, the differences between the intervention group and the control group were not statistically significant based on IL-35 and serum hs-CRP levels (*p* = 0.055 and *p* = 0.277, respectively). At the end of the study, there was no statistically significant difference between the two groups in terms of IL-35 (*p* = 0.172). However, the change levels of IL-35 between the two groups were statistically significant (*p* = 0.026). In terms of IL-35, in the intervention group, a statistically significant increase was seen when the data at the beginning and end of the study were compared (*p* = 0.031).

**Table 2:** Comparison of IL-35, hs-CRP and PAB levels between the two studies groups (intervention/control).

**Variable**	**Pomegranate (*n* = 32)**	**Control (*n* = 32)**	*P*-value*	***P*-value^£^**
IL-35 (ng/mL)				
Baseline	0.01 (0.0006, 0.03)	0.04 (0.01, 0.07)	0.055	0.091
Endpoint (8 weeks)	0.06 (0.02, 0.09)	0.04 (0.008, 0.08)	0.172	0.214
Changes	0.04 (0.003, 0.08)	0.0003 (-0.04, 0.04)	0.026	0.13
*P*-value**	0.031	0.832		
*P*-value^£^	0.077	0.832		
hs-CRP (mg/dL)				
Baseline	2.70 (1.12, 4.27)	3.94 (2.15, 5.72)	0.277	0.692
Endpoint (8 weeks)	2.85 (1.30, 4.41)	4.11 (2.36, 5.86)	0.167	0.835
Changes	0.15 (– 0.67, 0.98)	0.17 (– 1.17,1.52)	0.286	0.476
*P*-value**	0.331	0.464		
*P*-value^£^	0.413	0.464		
PAB (HK)				
Baseline	65.54 (53.01, 78.07)	63.57 (52.03, 75.12)	0.872	0.872
Endpoint (8 weeks)	64.37 (50.49, 78.25)	67.74 (57.25, 78.23)	0.401	0.668
Changes	– 1.17 (– 13.69, 11.35)	4.16 (– 3.57, 11.90)	0.274	>0.99
*P*-value**	0.852	0.285		
*P*-value^£^	>0.99	0.712		

Values are shown as mean (CI 95%).

*Reported *p*-value to compare the research variables between the groups (Mann–Whitney U test and independent sample T-test).

**Reported *p*-value to evaluate the research variables within the groups (Wilcoxon test and paired T-test).

^£^After correction.

In terms of serum hs-CRP levels, at the end of the study, no statistically significant differences were detected in the intra/intergroup comparison (*p* > 0.05).

**Figure 6:**
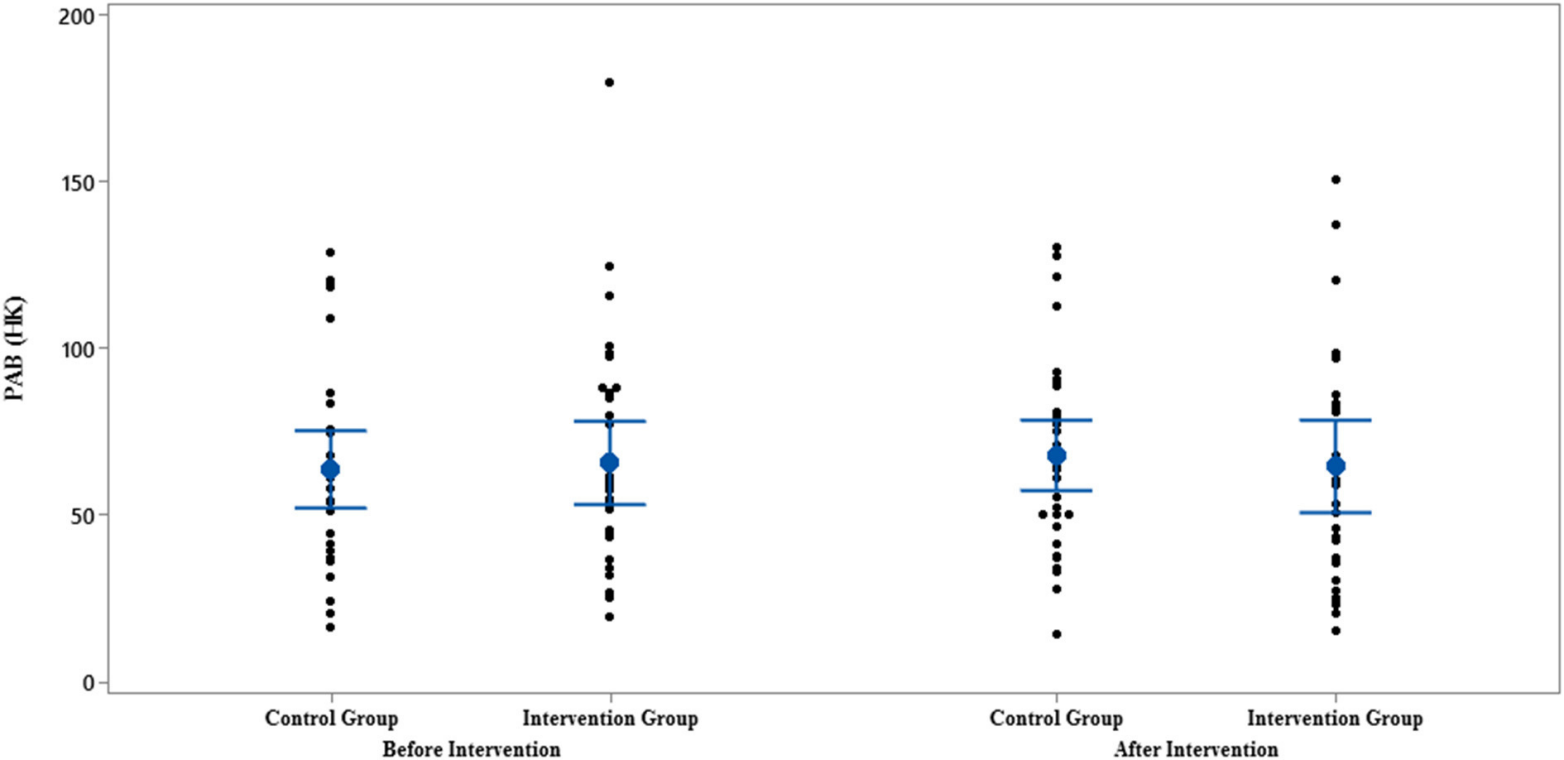
Interval plot of PAB pre- and post-intervention with 95% CI for the mean plus the dots for PAB values, in each group.

**Figure 7:**
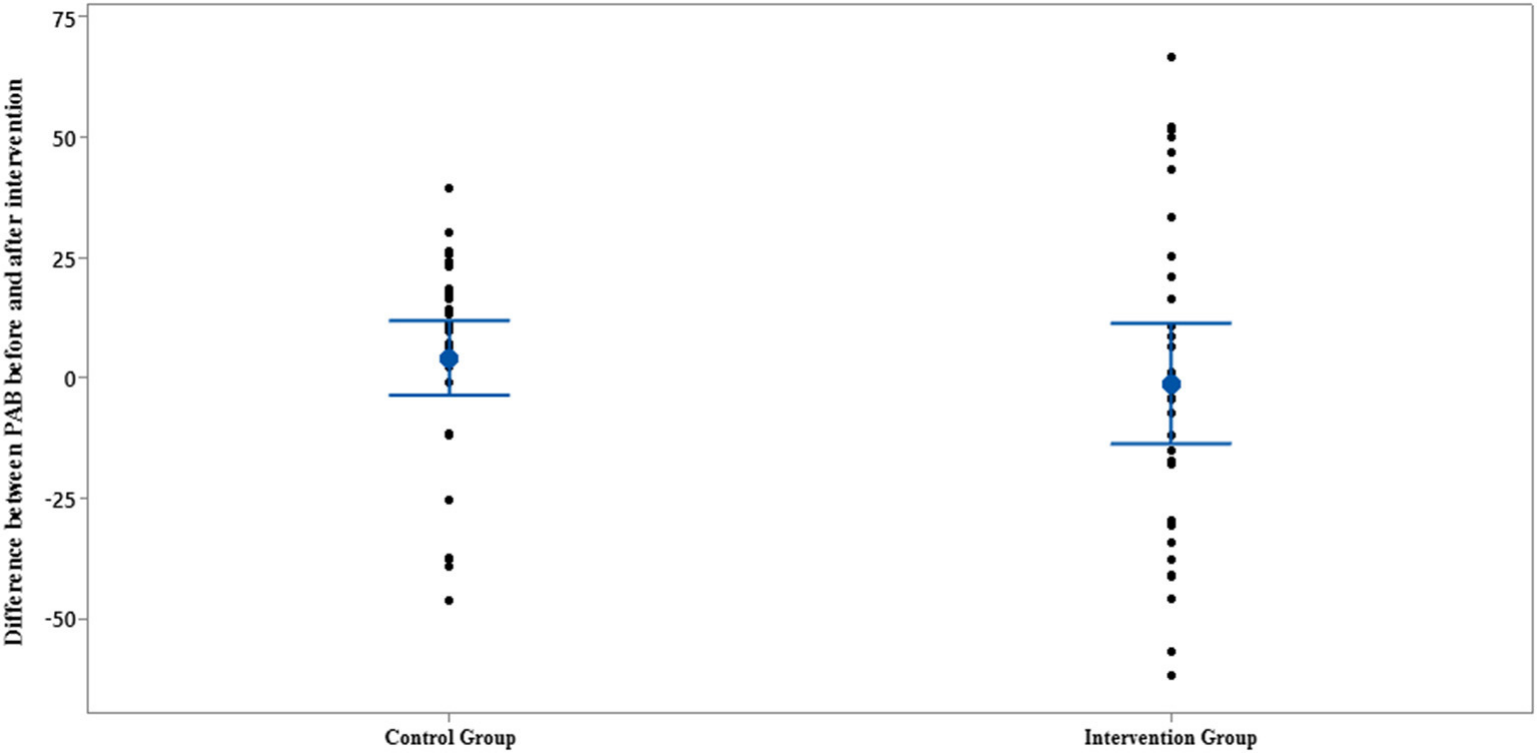
Interval plot of difference between PAB pre- and post-intervention with 95% CI for the mean plus the dots for PAB values, in each group.

The comparison of PAB in the control and intervention groups is presented in Figures 6, 7 and Table 2. As can be seen from Figures 6, 7 and Table 2, in terms of PAB, there were no statistically significant differences at the beginning and end of the study between the control and intervention groups (*p* = 0.872 and *p* = 0.401, respectively).

The results of subgroup analysis based on asthma severity showed that the changes in IL-35 were statistically significant only in the intervention group with mild asthma severity (*p* = 0.03). Also, the change levels of IL-35 were statistically significant in mild asthma severity between the intervention and control groups (*p* = 0.02). Regarding hs-CRP and PAB, no significant differences were observed in both mild and moderate asthma severity (*p* > 0.05) (Table 3).

**Table 3:** Comparison of IL-35, hs-CRP and PAB levels between the two studies groups (intervention/control).

**Variable**	**Pomegranate (*n* = 32)**	**Placebo (*n* = 32)**	*P*-value*
**Mild severity**			
IL-35 (ng/mL)			
Baseline	0.01 ± 0.00	0.06 ± 0.09	0.01
Endpoint (8 weeks)	0.05 ± 0.07	0.02 ± 0.05	0.38^£^
Changes	0.04 ± 0.07	−0.04 ± 0.12	0.02
*P*-value**	0.03	0.16	
hs-CRP (mg/dL)			
Baseline	1.75 ± 1.66	4.27 ± 6.59	0.73
Endpoint (8 weeks)	2.20 ± 3.71	3.44 ± 4.72	0.57
Changes	0.45 ± 2.69	0.45 ± 2.69	0.89
*P*-value**	0.37	0.46	
PAB (HK)			
Baseline	70.15 ± 42.12	65.58 ± 32.20	0.95
Endpoint (8 weeks)	63.38 ± 39.59	69.85 ± 33.43	0.53
Changes	– 6.77 ± 38.20	4.27 ± 21.04	0.4
*P*-value**	0.6	0.14	
**Moderate severity**			
IL-35 (ng/mL)			
Baseline	0.02 ± 0.07	0.02 ± 0.04	0.96
Endpoint (8 weeks)	0.06 ± 0.11	0.06 ± 0.13	0.5
Changes	0.04 ± 0.14	0.04 ± 0.14	0.39
*P*-value**	0.24	0.28	
hs-CRP (mg/dL)			
Baseline	3.64 ± 5.88	3.60 ± 2.64	0.32
Endpoint (8 weeks)	3.50 ± 4.90	4.78 ± 5.04	0.2
Changes	– 0.14 ± 1.85	1.18 ± 3.51	0.13
*P*-value**	0.53	0.12	
PAB (HK)			
Baseline	60.93 ± 25.99	61.57 ± 32.76	0.8
Endpoint (8 weeks)	65.36 ± 38.63	65.63 ± 24.93	0.59
Changes	4.43 ± 31.10	4.05 ± 22.59	0.55
*P*-value**	0.79	0.27	

Values are shown as mean ± standard deviation.

*Reported *p*-value to compare the research variables between the groups (Mann–Whitney U test or independent sample T-test).

**Reported *p*-value to evaluate the research variables within the groups (Wilcoxon test or paired T-test).

^£^Using ANCOVA test and adjustment for baseline value.

### Lung function

FEF_25 − 75%_, FEV_1_/FVC, FEV_1_, and FVC were examined at the beginning and end of the study. The results indicated that FEF_25 − 75%_, FEV_1_/FVC ratio, and FEV_1_ increased in the intervention group (*p* = 0.012, *p* = 0.001, and *p* = 0.031, respectively). Supplementation with pomegranate extract could only exert a statistically significant difference in FEV_1_/FVC ratio when comparing the control and intervention groups (*p* = 0.028). Additionally, the levels of change in the FEV_1_/FVC ratio between the two groups were statistically significant (*p* = 0.023) (Table 4).

**Table 4:** The comparison of changes in the spirometry tests between the intervention and control groups.

**Variable**	**Pomegranate (*n* = 32)**	**Control (*n* = 32)**	*P*-value*	***P*-value^£^**
FEV_1_ (%)				
Baseline	87.50 (81.18, 93.82)	84.75 (78.33, 91.17)	0.536	0.536
Endpoint (8 weeks)	92.94 (88.08, 97.80)	86.63 (81.63, 91.62)	0.069	0.172
Changes	5.43 (0.32, 10.55)	1.87 (– 3.58, 7.33)	0.199	0.331
*P*-value**	0.031	0.484		
*P*-value^£^	0.155	0.605		
FVC (%)				
Baseline	93.59 (87.76, 99.43)	94.38 (87.48, 101.27)	0.861	0.861
Endpoint (8 weeks)	96.81 (91.36, 102.27)	95.88 (90.60, 101.15)	0.802	>0.99
Changes	3.21 (-1.80, 8.24)	1.50 (– 2.63, 5.63)	0.375	0.937
*P*-value**	0.203	0.462		
*P*-value^£^	>0.99	0.77		
FEV_1_/FVC				
Baseline	96.16 (92.74, 99.57)	95.19 (90.24, 100.14)	0.957	0.957
Endpoint (8 weeks)	101.16 (97.77, 104.54)	94.43 (89.63, 99.24)	0.028	0.046
Changes	5.00 (2.19, 7.80)	– 0.75 (– 4.26, 2.76)	0.023	0.057
*P*-value**	0.001	0.661		
*P*-value^£^	0.005	0.826		
FEF_25 − 75%_				
Baseline	69.97 (58.98, 80.96)	69.66 (59.93, 79.38)	0.957	0.957
Endpoint (8 weeks)	78.34 (67.26, 89.43)	72.19 (62.43, 81.95)	0.46	0.766
Changes	8.37 (1.49, 15.25)	2.53 (-5.94, 11.00)	0.248	0.62
*P*-value**	0.012	0.541		
*P*-value^£^	0.06	0.676		

Values are shown as mean (CI 95%).

*Independent sample *T*-test.

**Paired *T*-test. ^£^After correction.

Based on the asthma severity, there were statistically significant changes in FEV_1_, FEV_1_/FVC, and FEF_25 − 75%_ in the intervention group with mild asthma severity (*p* = 0.02, *p* = 0.006, and *p* = 0.03, respectively). Also, the change levels in FEV_1_/FVC ratio were significant between the intervention and control groups in mild asthma severity (*p* = 0.008) (Table 5).

**Table 5:** The comparison of changes in the spirometry tests between the intervention and control groups.

**Variable**	**Pomegranate (*n* = 32)**	**Placebo (*n* = 32)**	*P*-value*
**Mild severity**			
FEV_1_ (%)			
Baseline	95.06 ± 14.48	93.75 ± 15.15	0.86
Endpoint (8 weeks)	98.56 ± 9.23	92.56 ± 13.49	0.22
Changes	3.50 ± 9.55	– 1.18 ± 17.89	0.17
*P*-value**	0.02	0.6	
FVC (%)			
Baseline	101.25 ± 11.27	99.88 ± 21.81	0.72
Endpoint (8 weeks)	101.81 ± 12.27	100.44 ± 15.81	0.95
Changes	0.56 ± 11.17	0.56 ± 15.00	0.65
*P*-value**	0.84	0.77	
FEV_1_/FVC			
Baseline	98.56 ± 9.94	99.06 ± 10.29	0.62
Endpoint (8 weeks)	104.00 ± 11.70	95.06 ± 13.89	0.05
Changes	5.43 ± 8.80	– 4.00 ± 9.34	0.008
*P*-value**	0.006	0.2	
FEF_25 − 75%_			
Baseline	77.38 ± 30.91	77.06 ± 19.48	0.66
Endpoint (8 weeks)	85.94 ± 36.59	75.06 ± 19.48	0.42
Changes	8.56 ± 14.61	−2.00 ± 22.71	0.11
*P*-value**	0.03	0.77	
**Moderate severity**			
FEV_1_ (%)			
Baseline	79.94 ± 17.41	75.75 ± 15.90	0.66
Endpoint (8 weeks)	87.31 ± 14.92	80.69 ± 11.78	0.2
Changes	7.37 ± 17.79	4.93 ± 11.56	0.57
*P*-value**	0.03	0.11	
FVC (%)			
Baseline	85.94 ± 17.00	88.88 ± 14.69	0.47
Endpoint (8 weeks)	91.81 ± 16.40	91.31 ± 12.13	0.98
Changes	5.78 ± 16.18	2.43 ± 6.68	0.31
*P*-value**	0.08	0.22	
FEV_1_/FVC			
Baseline	93.75 ± 8.60	91.31 ± 15.85	0.47
Endpoint (8 weeks)	98.31 ± 5.30	93.81 ± 13.16	0.09
Changes	4.56 ± 6.83	2.50 ± 9.28	0.49
*P*-value**	0.02	0.28	
FEF_25 − 75%_			
Baseline	62.56 ± 29.12	62.25 ± 31.52	0.98
Endpoint (8 weeks)	70.75 ± 22.16	69.31 ± 32.32	0.47
Changes	8.18 ± 23.23	7.06 ± 2412	0.95
*P*-value**	0.14	0.11	

Values are shown as mean ± standard deviation.

*Reported *p*-value to compare the research variables between the groups (Mann–Whitney *U* test or independent sample *T*-test).

**Reported *p*-value to evaluate the research variables within the groups (Wilcoxon test or paired *T*-test).

In moderate asthma severity, there were statistically significant changes in FEV_1_ and FEV_1_/FVC in the intervention group (*p* = 0.03 and *p* = 0.02, respectively). However, no statistically significant differences were observed between the two groups in terms of spirometry tests in moderate asthma severity (*p* > 0.05) (Table 5).

## Discussion

The effectiveness of ellagic acid, one of the main components of pomegranate, in alleviating allergic inflammation of the airways was shown in a mouse model (34). Asthma is an inflammatory illness that causes the lung cells to be overstimulated (35). It is characterized by increased airway responsiveness, inflammation, and structural changes. Many cytokines and other inflammatory mediators are released by immune system cells and airway structural cells, leading to respiratory tract inflammation in patients with asthma (5). Few studies have investigated the association between components of pomegranate and inflammatory factors. Bachoual et al. examined the influence of pomegranate peel extract on inflammation of lungs in mice. According to their results, pomegranate peel can inhibit myeloperoxidase (MPO) activity and thus decrease lung inflammation (36).

To the best of our knowledge, this study was the first double-blind, randomized trial on the impacts of pomegranate extract on lung function parameters, IL-35, hs-CRP, and PAB in patients with mild and moderate persistent allergic asthma. Our findings indicated that 500 mg/day of pomegranate supplementation ameliorated IL-35 and some spirometry test parameters in the intervention group.

At the end of the current research, a significant increase in IL-35, which has anti-inflammatory properties, was found in the intervention group. In 2007, IL-35 was recognized as an anti-inflammatory and immunosuppressive cytokine secreted by regulatory T cells (37). This interleukin acts on the original T cells, turning them into a family of regulatory T cells (T_reg_) called IL-35-producing T_reg_ (iT_R_35). These cells start to secrete IL-35. IL-35 decreases the immune response in allergic diseases, including asthma (37, 38). A study found that in mice whose expression of IL-35 was suppressed, the production of IL-17, hypersensitivity of the allergic airways, and the number of macrophages, neutrophils, and eosinophils were increased, probably indicating the anti-inflammatory role of this cytokine (39).

Wang et al. indicated that increased oxidative stress could induce apoptosis in T_reg_ cells (40). As previously mentioned, pomegranate is rich in antioxidants. As a result, pomegranate extract may effectively reduce the apoptosis of T_reg_ cells and increase IL-35 by reducing oxidative stress.

Evidence suggests that various mechanisms involve eosinophils in asthma exacerbation, including interaction with neutrophils (41). It has also been shown that ellagic acid effectively decreases the number of neutrophils and eosinophils in mouse models (42). In this study, it was also shown that pomegranate extract caused a significant decrease in the levels of changes in eosinophils and neutrophils in the intervention group compared to the control group (unpublished data).

Some fruits, including pomegranate, contain polyphenols such as ellagic acid and ellagitannin, and it was discovered that these fruits are rich in antioxidant and anti-inflammatory properties (43). Rasheed et al. found that pomegranate extract suppresses expression of pro-inflammatory cytokines in human cells by inhibiting nuclear factor (NF)-κB and mitogen-activated protein (MAP) kinase (44). Taheri Rouhi et al. proved that pomegranate juice and pomegranate seed could decrease inflammatory biomarkers such as tumor necrosis factor-α (TNF-α) and IL-6 in rats with diabetes mellitus (45).

As mentioned previously, supplementation with pomegranate extract did not significantly influence serum hs-CRP levels in the present study. A study on the impact of unsweetened pomegranate juice (240 mL) for 2 months on people with type 2 diabetes showed that pomegranate juice had no significant effect on serum hs-CRP levels (46). The findings of another study on the influences of pomegranate supplementation on the metabolic status of patients with type 2 diabetes proved that pomegranate supplementation did not exert a significant effect on serum hs-CRP levels (47). However, in a study on the impact of pomegranate juice (250 mL for 12 weeks) on inflammatory factors in patients with diabetes, Sohrab et al. showed that pomegranate juice consumption could significantly reduce serum hs-CRP levels (23). One possible mechanism of the influence of pomegranate on the reduction of serum hs-CRP levels is the expression suppression of pro-inflammatory cytokines by pomegranate, which is rich in polyphenols such as punicalagin and ellagic acid (44). One of the reasons for the difference between the result of Sohrab et al. and the current study may be the duration of the two studies (12 vs. 8 weeks). Despite significant changes in lung function parameters in the intervention group and significant changes in FEV_1_/FVC ratio between the two groups, no significant changes in serum hs-CRP were observed. A study showed no significant difference in serum hs-CRP levels in patients with different levels of asthma control based on GINA and FEV_1_. Additionally, it showed no relationship between the degree of systemic inflammation estimated by hs-CRP and other clinical indicators of asthma, such as FEV_1_ and GINA criteria (48). Therefore, asthma may be controlled, or spirometry tests may be improved, but the serum hs-CRP levels may not be changed. In the present study, as mentioned above, some spirometry tests improved in the intervention group, but hs-CRP levels did not show significant changes. These findings are supported by previous studies (49–51). Furthermore, the present study proved a small but significant change in IL-35, an anti-inflammatory factor, between the two groups; however, no significant change in serum hs-CRP levels was found. A study by Oflar et al. showed no significant correlation between IL-35 and serum hs-CRP (52).

Tannins and flavonoids are the other pomegranate compounds with antioxidant properties (53). Oxidative stress can trigger and exacerbate airway inflammation in patients with asthma (54). Our findings demonstrated that pomegranate supplementation (500 mg per day) for 8 weeks did not cause a significant decrease in the PAB values. Conversely, in terms of the within-group comparison, there was a decrease in PAB in the intervention group; however, it was not statistically significant. One reason for not observing a significant change in this parameter is that PAB may be an acute indicator of oxidative stress rather than a long-term indicator (32). Furthermore, Nobakht et al. demonstrated that the oxidation level is not related to the severity of the disease defined based on spirometry tests (55). To the best of our knowledge, no study has been conducted on the impact of pomegranate extract on PAB in people with asthma; however, in a study on the effect of 240 mL of sugar-free pomegranate juice on antioxidant factors in type 2 diabetes for 8 weeks, it was shown that the consumption of pomegranate juice had no significant effect on total antioxidant capacity (56). Conversely, a study on the impact of pomegranate juice (100 mL, 3 times/week for 1 year) on hemodialysis showed that it could reduce oxidative stress (57). According to the study mentioned above, long-term consumption of pomegranate can decrease oxidative stress.

As mentioned above, supplementation with pomegranate extract improved lung function parameters such as FEF_25 − 75%_, FEV_1_, and FEV_1_/FVC in patients with allergic asthma. Compared with the control group, supplementation only improved the FEV_1_/FVC ratio in the intervention group. In addition to FEV_1_, airway obstruction can be detected using a decrease in FEV_1_/FVC ratio (58). In this study, a significant increase in FEV_1_/FVC ratio in the intervention group indicates a reduction in airway obstruction. To date, no study has investigated the influence of pomegranate extract on spirometry tests in patients with asthma; however, a study showed that antioxidant vitamins such as vitamins C, E, and beta-carotene could affect lung health. The present study found that people in the lower quartiles of the serum antioxidant vitamins had less FEV_1_ and FVC compared to those in the upper quartiles in terms of these vitamins (59). Another study discovered that a daily intake of 40 mg more vitamin C was related to 25 mL more FEV_1_ and 23.3 mL more FVC (60). Dietary intake, as the primary source of non-enzymatic antioxidants, including vitamin C, seems to be correlated with a reduced risk of obstructive pulmonary disease, and an increase in vitamin C intake of 50 mg per day in men is associated with more FEV_1_ by 70.7 mL (61). Pomegranate contains antioxidants and is an excellent source of vitamin C, which is likely why it can improve spirometry test parameters.

Strengths of the present study included a randomized, double-blind, placebo-controlled trial design, the first randomized clinical trial to evaluate the effects of pomegranate extract in allergic asthma in a human model, and a stratified blocked randomization design based on asthma severity. The study duration was one of the limitations of the present study. Also, other inflammatory parameters such as TNF-α, IL-6, IL-4, IL-5 and IL-13 were not measured because of limited financial resources. Furthermore, we could not measure plasma polyphenol derivatives' levels of pomegranate extract in the participants.

## Conclusion

In conclusion, it seems that pomegranate extract can improve lung function parameters and IL-35 expression. To confirm the results of this study, it is recommended that studies with a longer duration should be conducted. It is also recommended that other inflammatory pathways (such as NF-κB, MAP kinase, and MPO activity) through which pomegranate extract may improve lung function parameters in asthma should be investigated.

## Data availability statement

The raw data supporting the conclusions of this article will be made available by the authors, without undue reservation.

## Ethics statement

The studies involving human participants were reviewed and approved by the Ethics Committee of Ahvaz Jundishapur University of Medical Sciences has approved this clinical trial (No. IR.AJUMS.REC.1398.905). The patients/participants provided their written informed consent to participate in this study.

## Author contributions

ZS and SH have made substantial contributions to conceptualize and to design the work. ZS and FA participated in the acquisition of data. SH was supervisor of this study. Medical experts of the current research were FA and MH. The statistical data were analyzed by EM. The manuscript was written by ZS and SH and edited by MZ. All authors contributed to the article and approved the submitted version.

## Funding

The sponsor of this study was Vice Chancellor for Research of Ahvaz Jundishapur University of Medical Sciences [grant number: NRC-9823]. This article is extracted from the Ph.D. thesis of the ZS of the present study.

## Conflict of interest

The authors declare that the research was conducted in the absence of any commercial or financial relationships that could be construed as a potential conflict of interest.

## Publisher's note

All claims expressed in this article are solely those of the authors and do not necessarily represent those of their affiliated organizations, or those of the publisher, the editors and the reviewers. Any product that may be evaluated in this article, or claim that may be made by its manufacturer, is not guaranteed or endorsed by the publisher.
